# Supplementary material for: BRAF Controls the Effects of Metformin on Neuroblast Cell Divisions in C. elegans
Source: Int J Mol Sci. 2020 Dec 26;22(1):178. doi: 10.3390/ijms22010178 (PMC7795703; doi:10.3390/ijms22010178)
Supplement: Supplementary file 1 [file ijms-22-00178-s001.pdf]

*aak-2(ok524)* worms fed with RNAi strains as descripted in the method in the main text. Control worms fed with empty vector strain: HT115α. The mix stage worms after fed were collected. Total RNA was extracted using High Pure RNA Isolation Kit (Roche). Five microgram of total RNA was used to synthesize cDNA using High Capacity cDNA Reverse Transcription Kit (Applied Biosystems). The qRT-PCR reactions were performed using Power SYBR Green PCR Master Mix (Applied Biosystems) and ABI 7500 system. The relative expression level of genes were carried out using $2^{-\triangle\triangle CT}$ method and normalized to *cdc-42* [1-4]

**Figure S1 qPCR results of target gene expression after fed with RNAi.**

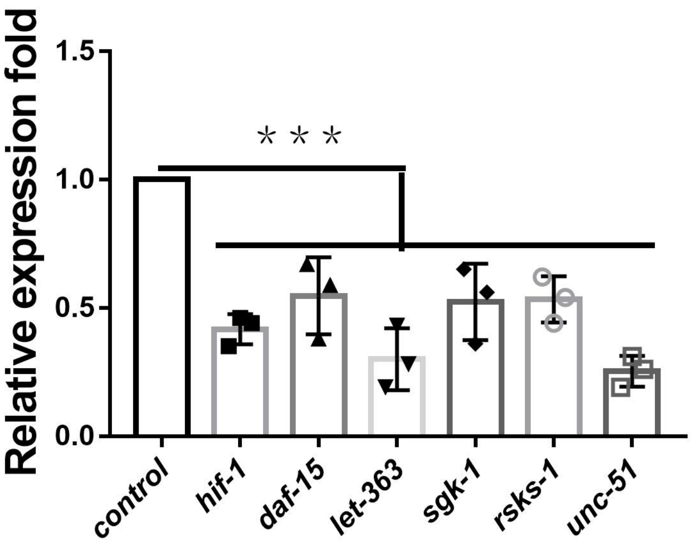


**Table S1 primers used in qPCR.**

| | |
|---|---|
| *cdc-42F* | 5'- CTGCTGGACAGGAAGATTACG-3' |
| *cdc-42R* | 5'- CTCGGACATTCTCGAATGAAG-3' |
| *let-363F* | 5'- TGCTATTATTATTCCGTTGTACAATCA -3' |
| *let-363R* | 5'- ATTTCGGAGCAGCTTCTGCGATTT -3' |
| *daf-15 F* | 5'- TGGCAGTTGATGTTGCCCGT-3' |
| *daf-15R* | 5'-AGTCGGGACATCCAGGAACAA-3' |
| *hif-1 F* | 5'- ACTTAACAGTCCCCCGAGGT-3' |
| *hif-1 R* | 5'- AGGCTTACGTCAATGTGCACAGGCCGAG-3' |
| *unc-51F* | 5'- TGCTTAACCGGACTGGATTC-3' |
| *unc-51 R* | 5'- CAATTTTCAGCCCCAAAACA-3' |
| *sgk-1 F* | 5'- ACATTCTTATTGGAACCAACCCT-3' |
| *sgk-1 R* | 5'- AATCATTTCAGCCTCCATTCTACTCA-3' |
| *rsks-1 F* | 5'- ATACAAAACCCACCTGAAAACCT-3' |
| *rsks-1 R* | 5'- CATGTCAAACATCAGAGCTCCGAGGCTCCAC-3' |

**Reference:**


1 Lee, S. J., Hwang, A. B. & Kenyon, C. Inhibition of respiration extends C. elegans life span via reactive oxygen species that increase HIF-1 activity. *Current biology : CB* **20**, 2131-2136, doi:10.1016/j.cub.2010.10.057 (2010).

2 Hsu, A. L., Murphy, C. T. & Kenyon, C. Regulation of aging and age-related disease by DAF-16 and heat-shock factor. *Science* **300**, 1142-1145, doi:10.1126/science.1083701 (2003).

3 Greer, E. L. *et al.* An AMPK-FOXO pathway mediates longevity induced by a novel method of dietary restriction in C. elegans. *Current biology : CB* **17**, 1646-1656, doi:10.1016/j.cub.2007.08.047 (2007).

4 Glauser, D. A., Johnson, B. E., Aldrich, R. W. & Goodman, M. B. Intragenic alternative splicing coordination is essential for Caenorhabditis elegans slo-1 gene function. *Proceedings of the National Academy of Sciences of the United States of America* **108**, 20790-20795, doi:DOI 10.1073/pnas.1116712108 (2011).
